# Analysis of merged whole blood transcriptomic datasets to identify circulating molecular biomarkers of feed efficiency in growing pigs

**DOI:** 10.1186/s12864-021-07843-4

**Published:** 2021-07-03

**Authors:** Farouk Messad, Isabelle Louveau, David Renaudeau, Hélène Gilbert, Florence Gondret

**Affiliations:** 1 PEGASE, INRAE, Institut Agro, 35590 Saint-Gilles, France; 2 GenPhySE, INRAE, INP-ENVT, 31326 Castanet Tolosan, France

**Keywords:** Biomarkers, Blood, Feed efficiency, Gradient TreeNet boosting, Microarray, Random Forest, Residual feed intake

## Abstract

**Background:**

Improving feed efficiency (FE) is an important goal due to its economic and environmental significance for farm animal production. The FE phenotype is complex and based on the measurements of the individual feed consumption and average daily gain during a test period, which is costly and time-consuming. The identification of reliable predictors of FE is a strategy to reduce phenotyping efforts.

**Results:**

Gene expression data of the whole blood from three independent experiments were combined and analyzed by machine learning algorithms to propose molecular biomarkers of FE traits in growing pigs. These datasets included Large White pigs from two lines divergently selected for residual feed intake (RFI), a measure of net FE, and in which individual feed conversion ratio (FCR) and blood microarray data were available. Merging the three datasets allowed considering FCR values (Mean = 2.85; Min = 1.92; Max = 5.00) for a total of *n* = 148 pigs, with a large range of body weight (15 to 115 kg) and different test period duration (2 to 9 weeks). Random forest (RF) and gradient tree boosting (GTB) were applied on the whole blood transcripts (26,687 annotated molecular probes) to identify the most important variables for binary classification on RFI groups and a quantitative prediction of FCR, respectively. The dataset was split into learning (*n* = 74) and validation sets (*n =* 74). With iterative steps for variable selection, about three hundred’s (328 to 391) molecular probes participating in various biological pathways, were identified as important predictors of RFI or FCR. With the GTB algorithm, simpler models were proposed combining 34 expressed unique genes to classify pigs into RFI groups (100% of success), and 25 expressed unique genes to predict FCR values (*R*^2^ = 0.80, RMSE = 8%). The accuracy performance of RF models was slightly lower in classification and markedly lower in regression.

**Conclusion:**

From small subsets of genes expressed in the whole blood, it is possible to predict the binary class and the individual value of feed efficiency. These predictive models offer good perspectives to identify animals with higher feed efficiency in precision farming applications.

**Supplementary Information:**

The online version contains supplementary material available at 10.1186/s12864-021-07843-4.

## Background

Peripheral blood is widely used in human medicine and veterinary fields as a relevant and easy sampling source of biological information, since it transports a large variety of molecules including DNA, coding and non-coding regulatory RNA, proteins and metabolites from all over the body. Their dynamics reflects homeostatic regulation [1–3], physiological changes [4, 5] and variations in immune capacity [6, 7]. Circulating molecules also provide valuable insights into complex phenotypes such as obesity and diabetes [8, 9], health status [10], sensitivity to heat stress [11] and nutrient efficiency for productive outputs [3, 7, 12]. Therefore, they hold much promise for the identification of biomarkers for particular phenotype prediction [13]. Both hypothesis-based and discovery-based procedures are used for the search of biomarkers. For a discovery-based procedure, high-throughput expression studies analyzed by linear model statistics and functional annotation bioinformatics are often used to enlighten how expressed genes and related biological pathways are discriminants between treatments. However, a plethora of machine learning (ML) approaches applied on data gathered in a learning base from characterized samples have the potential to surpass these traditional approaches in predicting class membership and individual values of unknown samples gathered in a test base [14]. In conditions where small variations in the data may cause significant changes in the prediction, these methods generally overcome complex, noisy and hidden relationships when ranking the most important genes for prediction and avoid the pitfalls of overfitting.

Feed efficiency (FE) has become a research priority in growing pigs to support competitive and sustainable meat production. Improving FE is a strategy to reduce the amount of feed needed to produce meat and to reduce environmental wastes and emissions. Feed efficiency is measured on a farm as feed conversion ratio (FCR), calculated as the ratio of an amount of feed intake to body weight (BW) gain. Residual feed intake (RFI) has also been proposed as a refined measure of net FE in selection experiments [15]. It is defined as the difference between the observed feed intake and the feed intake predicted from growth and maintenance requirements. For RFI, BW gain and indicators of body composition such as backfat thickness must be recorded during a test period for each animal. This is time-consuming and costly, especially when animals are housed in group. Moreover, FE is underlined by variations in the transcripts of several genes participating in many functional pathways in different tissues [16], which adds to its complexity. Therefore, there is a need to find molecular biomarkers that accurately differentiate high and low FE animals and that can be further used for improving FE of growing animals in breeding programs or nutritional decision tools. So far, various studies have revealed differences in the whole blood transcriptome between low RFI (most feed efficient) and high RFI pigs (less feed efficient) at post-weaning [12] and during the growing period [3, 7]. Moreover, the concentration of IGF-1 in blood plasma of juvenile post-weaned pigs was correlated with RFI measured during the growing period [17], suggesting that circulating molecules may even serve as early indicators for FE. However, among genes identified as differentially expressed between steers with low or high BW gain and feed intake, only few of them were similarly found across different cohorts [18]. This highlights the importance of incorporating different datasets to cover various experimental conditions and to avoid the limits of each design (number of samples/number of treatments) for biomarker discovery.

This study aimed to identify reliable sets of expressed genes in the whole blood to predict the RFI group or individual FCR value. For that, ML algorithms were applied on a merged transcriptomic dataset from three independent experiments where meta-data for RFI and FCR were also available in growing pigs.

## Results

### Animals and FE traits

**Fig. 1:**
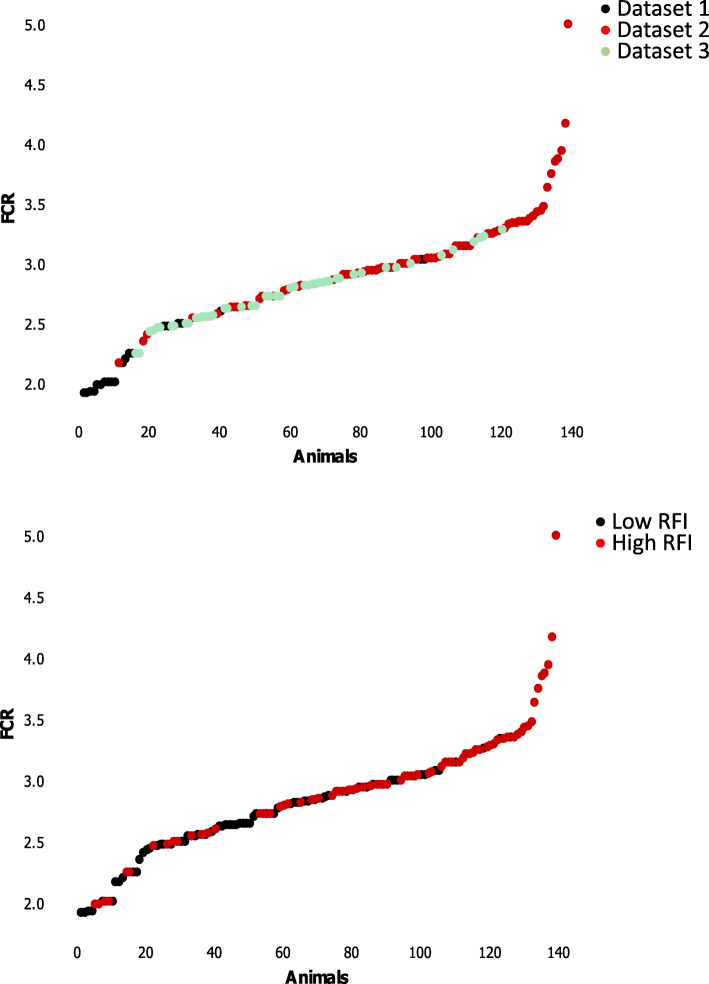
Distribution of feed conversion ratio across the dataset. Pigs of low and high residual feed intake (RFI) lines were considered in three different experiments unraveling different periods for blood sampling. Feed conversion ratio (FCR) was measured for each pig during specific test periods. The first dataset included 21 pigs, the second dataset included 48 pigs and the third dataset included 79 pigs. In the merged dataset, 148 pigs were thus analyzed. Feeding conditions, test periods and age and body weight of pigs when blood sampling was performed, are detailed in Material and Methods

Three independent experiments [19–21] were merged to reanalyze gene expression levels in whole blood from a total of 148 females and barrows. These experiments all included purebred French Large White pigs of two lines divergently selected for RFI during 7th to 9th generations, and were based on different dietary treatments. The distribution of FCR values for the 148 pigs considered in the merged dataset was illustrated in Fig. 1, according to the RFI group and their experiment of origin. The FCR averaged 2.85 kg feed/kg BW, and covered a large range of values (Min = 1.92; Max = 5.00). It was generally lower for pigs of the low RFI line than for pigs of the high RFI line, but there was an interpenetration between the two lines within each experiment and between experiments.

### Model performance in RFI classification

Merging the transcriptomic data of the three independent experiments resulted in a new dataset of 26,687 annotated expressed probes across the 148 blood samples. The random forest (RF) and gradient tree boosting (GTB) procedures were applied to this merged dataset to find the most important transcripts that allow the classification of pigs (low RFI/high RFI). These algorithms were considered to produce an excellent fit of predicted to observed values even when the specific nature of the relationships between the predictor variables and the dependent variable was very complex [22]. In the two procedures, a randomly selected bootstrap sample set was used as a learning dataset (*n =* 74 pigs), whereas the remaining samples (*n =* 74 pigs) were used in a test dataset for validation. Learning and validation datasets including transcriptomic data and meta-data (RFI group, FCR) are freely available at https://doi:10.15454/J4XOPD.

From the RF procedure, a total of 778 probes (out of the 26,687 annotated probes) were first selected to provide an accurate classification of pigs into low and high RFI groups during the training step. In the validation step (Supp. Table S1), the RF model further selected 328 probes (out of the 778 probes) as very important variables (VIP) for RFI classification. The accuracy of the model was estimated by the proportion (%) of good classification, and the optimal model was selected according to the receiver operating characteristic curve (ROC) as a diagnostic ability of the binary classifier system. Iterative steps allowed to obtain the best model (96% of success on average) with a subset of 50 molecular probes (out of the 328 VIP). It provided a good prediction for 94.74% of the high RFI pigs and 97.22% of the low RFI pigs, respectively (Table 1), so that the prediction accuracy was similar for the two RFI lines (chi2 = 0.59). The 50 VIP corresponded to 25 unique identified expressed genes since 17 probes had no consolidated annotation and some genes were represented by two up to four probes (*GPX3, CD1A,* and *SERPINF1*). The list of these 50 probes, the encoded genes, and the score attributed to each probe in the predictive RF model is given in Supp. Table S2.

**Table 1:** Classification of pigs between RFI groups based on 50 molecular probes expressed in blood

Actual class	Nb pigs	Percent correct	Predicted classes	
			High RFI	Low RFI
**Random Forest procedure**				
High RFI	38	94.7%	36	2
Low RFI	36	97.2%	1	35
Total	74			
Overall %Correct		96.0%		
**Gradient Tree Boosting procedure**				
High RFI	38	100%	38	0
Low RFI	36	100%	0	36
Total	74			
Overall %Correct		100%		

Random forest (RF) and gradient treenet boosting (GTB) algorithms were applied on transcriptomic dataset from the whole blood sampled from 148 pigs of lines divergently selected for residual feed intake (RFI). Pigs were randomly split into training (*n* = 74) and validation test (*n* = 74) datasets to evaluate model performance in classifying pigs into low or high RFI groups. Expression levels of 50 molecular probes were considered in the validation set. The model made no error (100% of success) when built by GTB procedure

From the GTB procedure, a total of 728 probes (out of the 26,687 annotated probes) were similarly retained as providing an accurate classification on low/high RFI during the training step. In the validation step, the GTB model further identified 391 probes (out of 728 probes) as the best VIP to classify pigs on low or high RFI (Supp. Table S1). Iterative steps led to select a subset of 50 molecular probes (out of the 391 VIP) allowing 100% of good classification (Table 1). These 50 probes corresponded to 34 unique annotated expressed genes (Table 2); these genes were all represented by a single probe in the model but 16 probes had no consolidated annotation. The top five transcripts for classifying pigs into low or high RFI were the following: *PSEN1*, *SERPINF1*, *TMEM63B*, *EPAS1* and *MX1* genes (Table 2).

**Table 2:** List of blood genes retained as very important to classify pigs for RFI^1^

Probe name	Gene symbol	Full name	Score
A_72_P304024	PSEN1	presenilin 1	100
A_72_P008221	SERPINF1	serpin peptidase inhibitor, clade F (alpha-2 antiplasmin, pigment epithelium der	87.7
A_72_P047696	TMEM63B	transmembrane protein 63B	60.8
A_72_P035801	EPAS1	endothelial PAS domain protein 1	59.9
A_72_P010326	MX1	myxovirus (influenza virus) resistance 1, interferon-inducible protein p78 (mous	59.2
A_72_P359418	WDHD1	WD repeat and HMG-box DNA binding protein 1	57.4
A_72_P418319	HTRA1	HtrA serine peptidase 1	56.2
A_72_P201717	NPR3	natriuretic peptide receptor C/guanylate cyclase C (atrionatriuretic peptide rec	56.1
A_72_P061216	ADAM9	ADAM metallopeptidase domain 9	54.5
A_72_P548816	HMG20A	high mobility group 20A	51.9
A_72_P035056	BCO2	beta-carotene oxygenase 2	50.7
A_72_P183616	TEX2	testis expressed 2	50.1
A_72_P039066	EIF1B	eukaryotic translation initiation factor 1B	48.4
A_72_P036051	GPX3	glutathione peroxidase 3 (plasma)	47.0
A_72_P131741	SLC46A3	solute carrier family 46, member 3	46.2
O12841	PARVG	parvin, gamma	42.8
A_72_P001891	SPTLC2	serine palmitoyltransferase, long chain base subunit 2	42.5
A_72_P250342	RPS18	ribosomal protein S18	40.8
O8712	ENO3	enolase 3 (beta, muscle)	39.7
A_72_P094676	UGCG	UDP-glucose ceramide glucosyltransferase	39.2
A_72_P051041	MKI67	antigen identified by monoclonal antibody Ki-67	38.6
A_72_P128591	SCML1	sex comb on midleg-like 1 (Drosophila)	38.5
A_72_P002751	JPH4	junctophilin 4	38.3
A_72_P200892	ZNF672	zinc finger protein 672	33.1
A_72_P177616	DCT	dopachrome tautomerase (dopachrome delta-isomerase, tyrosine-related protein 2)	32.6
A_72_P619999	OAZ3	ornithine decarboxylase antizyme 3	32.3
A_72_P134026	NUP43	nucleoporin 43 kDa	30.2
A_72_P126346	WBSCR27	Williams Beuren syndrome chromosome region 27	30.1
A_72_P000776	PAG1	phosphoprotein associated with glycosphingolipid microdomains 1	29.7
A_72_P185296	CLU	Clusterin	29.3
A_72_P289839	ZNF3	zinc finger protein 3	27.4
A_72_P470830	ORC4	origin recognition complex, subunit 4	27.4
A_72_P000506	CREBRF	CREB3 regulatory factor	27.9
A_72_P499239	TSPAN7	tetraspanin 7	16.3

^1^A gradient tree boosting (GTB) algorithm was applied on transcriptomic dataset (26,687 annotated molecular probes) from the whole blood of 148 growing pigs. Data were split into training (*n =* 74) and validation test (*n =* 74) subsets to evaluate model performance in classifying pigs into low or high residual feed intake (RFI) groups. The unique genes corresponding to the most relevant annotated probes able to attribute RFI class for each pig were listed. The score attributed to each probe gave hierarchy of importance in the predictive model

Overall, 12 annotated expressed genes (25% of the genes retained by each method) were commonly proposed by the RF and GTB models as top VIPs to classify pigs on low or high RFI. They were *PSEN1, SERPINF1, EPAS1, GPX3, CLU, WDHD1, HTRA1, PARVG, HMG20A, RPS18, SLC46A3* and *DCT*.

### Model performance in FCR prediction

**Fig. 2:**
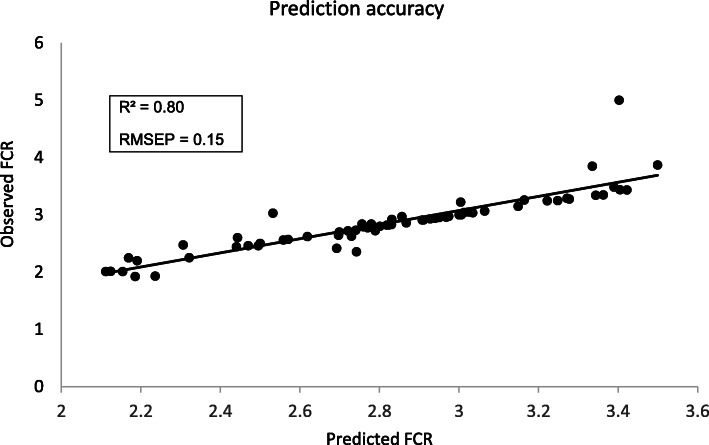
Regression analysis of the relationship between observed and predicted FCR. A predictive model to identify the most important annotated expressing probes able to predict feed-conversion-ratio (FCR) was built from the whole blood transcriptome merged from three independent experiments, and using a Gradient TreeNet Boosting (GTB) algorithm. Randomly selected bootstrap pig samples (*n* = 74) were used for learning, whereas the remaining samples (*n =* 74) were used for validation. Iterative steps led to retain a set of 50 very important variables. The graph was then computed between observed and predicted FCR values. Accuracy of the prediction was estimated by using R squared (*R*^2^) and root mean square error of prediction (RMSEP). Pigs considered in the study were from two divergent selection lines for residual feed intake (RFI), a measure of net feed efficiency. The red square represents pigs of the high RFI line, and the blue dot represents pigs of the low RFI group. No specific bias in prediction was observed due to RFI line

When addressing continuous variables such as FCR, regression trees rather than classification trees must be built. The transcriptomic dataset was similarly split into training (*n* = 74) and validation (*n =* 74) datasets. About 1393 probes (out of the 26,687 annotated probes) were selected during the training procedure. The performance of the models was then evaluated by using the validation set, and models with the best *R*^2^ and the lowest Root Mean Squared Error (RMSE) were retained (Table 3). The accuracy of prediction by the GTB algorithm (*R*^2^ ~ 0.80; RMSE ~ 0.23) exceeded that obtained by the RF procedure (*R*^2^ ~ 0.65 and RMSE ~ 0.29). Due to this large difference in model performance between the two algorithms in regression, also mentioned by others [23], only the results of the GTB model for FCR prediction were described in this study. The GTB procedure first identified 428 probes as a top VIP to predict FCR values. Iterative steps led to a good compromise between a lower number of VIP and increased accuracy of the prediction, which was obtained with 50 molecular expressed probes. These 50 probes corresponded to 27 unique annotated genes (Table 4). Finally, the predicted (X) values were compared with the observed (Y) values for the pigs included in the validation set (*n* = 74). The quality of the relationships was evaluated based on the RMSE of prediction (RMSEP) obtained by a leave-one-out cross-validation from the value of the predicted residual sum of squares. Observed and predicted values for FCR were very close (*R*^2^ = 0.80, RMSEP = 0.15; Fig. 2). The mean of predicted FCR values was 2.83 and the mean for observed FCR values was 2.85, respectively, and the error made by the model was evaluated at 7% on the average. The samples (*n* = 5) having the highest residual (> 0.15) all corresponded to pigs of the high RFI line but from different experiments (1 pig from experiment 1, 1 pig from experiment 2, and 3 pigs from experiment 3, Supp. Fig. S1), suggesting no bias due to the independent datasets. Without these few samples (5 out of 74), the prediction accuracy was improved (*R*^2^ = 0.94).

**Table 3:** Iterative steps for model reduction to predict FCR values^1^

	Number of probes	Number of genes	*R*^2^	RMSE
**Random Forest procedure**				
FCR	604	411	0.42	0.366
	100	58	0.62	0.301
	**50**	**30**	**0.65**	**0.293**
	25	17	0.67	0.281
	10	8	0.68	0.278
**Gradient Tree Boosting**				
FCR	728	477	0.78	0.241
	100	56	0.79	0.235
	**50**	**27**	**0.80**	**0.234**
	25	12	0.81	0.229
	10	5	0.80	0.223

Random forest (RF) or gradient treenet boosting (GTB) algorithms were applied on a transcriptomic dataset containing 26,687 molecular probes measured in whole blood sampled from 148 pigs. Dataset was split into training (*n =* 74) and validation test (*n* = 74) subsets to evaluate model performance in predicting food conversion ratio (FCR). The first rounds led to model stabilization with 604 molecular probes as very important variables (VIP) for FCR prediction using RF and 728 probes for FCR prediction with GTB, respectively, out of the 26,687 expressed annotated probes. The second entry was an iterative step of the former procedure, but considering the VIP identified in the first step as the new inputs. This increased the accuracy of the prediction evaluated by the root mean square error (RMSE) and the coefficient of determination (*R*^2^). Iterative steps were further performed. The numbers of annotated probes and their corresponding unique genes identified as VIP were indicated at each step. Iterative models were almost equivalent in performance, so that the ones including 27–30 unique genes were further selected. Models obtained with GTB algorithms performed better than those obtained by using RF procedures

**Table 4:** List of blood genes identified as very important in FCR prediction^a^

Probe name	Gene symbol	Full name	Score
A_72_P004376	SLC36A4	solute carrier family 36 (proton/amino acid symporter), member 4	100.00
A_72_P052096	SEPTIN6	septin 6	88.24
A_72_P035551	PSMB9	proteasome (prosome, macropain) subunit beta type, 9	77.53
A_72_P006596	GNG12	guanine nucleotide binding protein (G protein), gamma 12	75.40
A_72_P441179	KLF1	Kruppel-like factor 1 (erythroid)	74.39
A_72_P027206	CCDC70	coiled-coil domain containing 70	73.21
A_72_P000681	IRF2BP2	IRF2 binding protein 2	70.00
A_72_P155326	IGF2	insulin growth factor 2	69.17
A_72_P000006	ZNF644	zinc finger protein 644	68.67
A_72_P001306	AAGAB	alpha- and gamma-adaptin binding protein	68.31
A_72_P008086	SLC39A9	solute carrier family 39 (zinc transporter), member 9	67.00
A_72_P000171	SHPRH	SNF2 histone linker PHD RING helicase, E3 ubiquitin protein ligase	65.24
A_72_P005536	DIAPH3	diaphanous homolog 3 (Drosophila)	64.94
A_72_P001051	FCRLA	Fc receptor-like A	63.55
A_72_P000371	SDR39U1	short chain dehydrogenase/reductase family 39 U member 1	63.36
A_72_P001061	CD84	CD84 molecule	61.94
A_72_P001366	MORC2	MORC family CW-type zinc finger 2	61.81
A_72_P010816	MMAA	methylmalonic aciduria (cobalamin deficiency) cblA type	61.38
A_72_P000376	TRIM38	tripartite motif containing 38	61.12
A_72_P001201	FEM1C	fem-1 homolog c (C. elegans)	59.74
A_72_P023626	NUAK1	NUAK family, SNF1-like kinase, 1	56.91
A_72_P000856	TRIM46	tripartite motif containing 46	53.82
A_72_P002226	GEMIN5	gem (nuclear organelle) associated protein 5	51.67
A_72_P043191	PIKFYVE	phosphoinositide kinase, FYVE finger containing	51.53
A_72_P000356	MACF1	microtubule-actin crosslinking factor 1	51.07
A_72_P614951	SEPP1	selenoprotein P, plasma, 1	47.05
A_72_P021346	RBM25	RNA binding motif protein 25	43.75

^a^A gradient treenet boosting (GTB) algorithm was applied on transcriptomic dataset (26,687 molecular probes) from the whole blood of 148 growing pigs. Data were split into training (*n =* 74) and validation test (*n =* 74) subsets to evaluate model performance in classifying pigs into low or high residual feed intake (RFI) groups. The unique genes corresponding to the most relevant annotated probes able to predict feed conversion ratio (FCR) for each pig were listed. The score attributed to each probe gave hierarchy of importance in the predictive model

### Overview of the biological pathways shared by the molecular predictors of RFI and FCR traits

To progress in the knowledge of the most important biological pathways underlying the variation of FE among pigs, the expressed genes selected by the GTB models as top VIP allowing binary diagnostic for RFI (low/high) or predicting FCR (individual values) were submitted to a functional analysis using n bioinformatics tool. The 391 molecular probes initially selected to split pigs into low and high RFI groups, corresponded to 253 annotated unique genes that were clustered into 14 biological pathways (Table 5). The lipid metabolic process and transport, response to oxidative stress, phosphorylation, and positive regulation of defense response were among the top functional pathways identified across these genes. The 728 molecular probes selected to predict FCR values corresponded to 477 unique annotated genes that were clustered in 10 biological pathways (Table 5). Significant pathways were related to immune and defense response (regulation of leukocyte activation, regulation of cytokine production, regulation of acute inflammatory response and positive regulation of immune response), glycoprotein metabolic process, regulations of protein transport and of peptidase activity, and protein amino acid auto-phosphorylation.

**Table 5:** Main overrepresented biological processes shared by genes selected as predictors of feed efficiency traits

GO Terms	Nb genes	E	PValue	Clustered genes
**Clustered pathways among 391 probes corresponding to 253 unique genes first selected to classify pigs on low/high RFI**				
GO:0006643 ~ membrane lipid metabolic process	6	1.93	0.004	TEX2, SPTLC2, PSAP, COL4A3BP, UGCG, SMPD3
GO:0006979 ~ response to oxidative stress	8	1.66	0.006	PLA2G4A, PSEN1, EPAS1, CLU, GPX3, JAK2, ADAM9, DHCR24
GO:0006869 ~ lipid transport	8	1.59	0.003	OSBPL3, PSAP, COL4A3BP, CLU, PCTP, ABCA1, APOM, CROT
GO:0016310 ~ phosphorylation	20	1.55	0.010	IRAK2, FCER1A, ND2, TGFBR1, BMPR2, EIF2A, ULK4, GALK2, NDUFV3, VRK1, PSEN1, GCK, COL4A3BP, TGFBR3, JAK2, ATP5O, CIT, THBS1, MYLK, ADAM9
GO:0031349 ~ positive regulation of defense response	6	1.34	0.003	FCER1A, IRAK2, PLA2G4A, CADM1, IL6ST, JAK2
GO:0000267 ~ cell fraction	23	1.33	0.036	JPH4, CADM1, CYP51A1, SLC22A7, UGCG, HPS1, CCDC47, ATP1A1, NMB, ABCA1, NPR3, IL15, KARS, DCT, JUP, PLA2G4A, PSEN1, GCK, GPX3, SRR, ENO3, JAK2, ACSL3
GO:0009725 ~ response to hormone stimulus	10	1.27	0.055	PLA2G4A, ENPP1, SOCS3, TGFBR1, TGFBR3, JAK2, PIK3R3, THBS1, BRCA1, ADAM9
GO:0008361 ~ regulation of cell size	5	1.25	0.295	ENPP1, TGFBR1, SMAD4, TGFBR3, NTN1
GO:0030278 ~ regulation of ossification	4	1.16	0.085	PLA2G4A, ENPP1, IL6ST, BMPR2
GO:0017015 ~ regulation of transforming growth factor beta receptor signaling pathway	4	1.09	0.015	HTRA1, CHST11, SMAD4, THBS1
GO:0051091 ~ positive regulation of transcription factor activity	4	1.05	0.045	IRAK2, UBE2V1, TGFBR3, JAK2
GO:0042470 ~ melanosome	5	1.03	0.033	DCT, STOM, SERPINF1, RAB35, ATP1A1
GO:0007498 ~ mesoderm development	4	1.02	0.075	MACF1, BMPR2, EOMES, JAK2
**Clustered pathways among 728 probes corresponding to 477 unique genes first selected to predict FCR**				
GO:0002694 ~ regulation of leukocyte activation	13	1.89	0.002	CD83, CD86, CD80, STAT5A, IL27, IL4R, IL1B, CD4, IL15, CD40, PAG1, THY1, SYK
GO:0009100 ~ glycoprotein metabolic process	14	1.79	0.003	ATP7A, B3GNT9, MGAT4A, GALNT1, TRAK2, HPSE, CHST11, ACAN, CD4, FUT1, OGT, UGGT2, ST6GALNAC2, DHCR24
GO:0001817 ~ regulation of cytokine production	15	1.65	0.000	CADM1, PANX1, IGF2, STAT5A, IL27, CD40, NLRP3, DDX58, CD83, CD86, CD80, IL1B, CD4, CLEC7A, SYK
GO:0051223 ~ regulation of protein transport	8	1.65	0.034	CADM1, PANX1, IGF2, ANG, IL1B, CD40, NLRP3, DNAJC1
GO:0002673 ~ regulation of acute inflammatory response	5	1.51	0.002	PLA2G4A, C3, IGF2, SERPING1, CCL5
GO:0052547 ~ regulation of peptidase activity	7	1.39	0.028	SLC11A2, CYCS, BCL2L13, HBXIP, NLRP3, EIF2AK3, DHCR24
GO:0046777 ~ protein amino acid autophosphorylation	6	1.32	0.079	FYN, CLK4, KIT, LRRK2, EIF2AK3, SYK
GO:0050778 ~ positive regulation of immune response	10	1.15	0.017	CADM1, C3, FYN, STAT5A, IL1B, SERPING1, IL15, CLEC7A, THY1, SYK
GO:0031349 ~ positive regulation of defense response	6	1.01	0.047	PLA2G4A, CADM1, C3, STAT5A, CLEC7A, CCL5
GO:0032881 ~ regulation of polysaccharide metabolic process	3	1.01	0.060	PPP1R3C, ENPP1, IGF2

Very important genes for prediction of feed efficiency traits (RFI: residual feed intake; FCR: feed conversion ratio) were clustered into functional groups using DAVID tool. The enrichment score (E > 1) for each cluster and *P*-value of the enrichment for the corresponding Gene Ontology (GO) terms are provided. Iterative steps for model reduction have been further applied on these transcripts of genes to obtain smaller sets of predictors

The subset of the 50 best VIP to predict FCR values are participating in a variety of pathways, such as the regulation of immune system response (*CD84, IGF2, PSMB9, TRIM38, PIKFYVE, KLF1, IRF2BP2*), protein metabolism and especially ubiquitination process (*SHPRH, PSMB9, FEM1C, TRI*M38), response to peptides and organic substances (*GNG12, KLF1, IGF2, PSMB9, TRIM38*), lipid and cholesterol metabolic process (*MORC2 CYP51A1, DHCR24*), oxido-reduction (S*DR39U1*) or intracellular transport (*CD84, GEMIN5, PIKFVE, AAGAB, SLC36A4*). This suggested equal importance of many biological routes in the variation of FCR, and underlined genes as pivots in inter-related pathways.

Overall, 63 unique genes (i.e., 8% of all VIPs) expressed in the whole blood were identified as common VIP for the two FE traits (Table 6). Among them, *BCO2, CREBRF, GPX3, HMG20A, JPH4, PAG1* and *SPTLC2* were notably included in the list of top 50 VIP for RFI, while *IRF2BP2, MACF1, MORC2, SDR39U1, TRIM46* and *ZNF644* were included in the list of top 50 VIP for FCR.

**Table 6:** List of the 63 blood genes identified as common predictors for two feed efficiency traits

Traits	Common VIP^a^
**RFI/FCR**	ADAP2; APCDD1; ARHGEF10L; ARRDC3; BCO2; CADM1; C6orf89; CHST11; CIT; CREBRF; CROT; CYHR1; CYP51A1; DHCR24; EIF2A; ENPP1; ESCO1; FAF2; GIMAP8; GPX3; HMG20A; HOXD3; IL15; IGF2; IRF2BP2; JPH4; KCNH2; MACF1; MORC2; NT5DC3; P2RY1; PAG1; PHKB; PLA2G4A; PLXNC1; PPCDC; PSAP; RBM38; RPS17; SCUBE3; SDR39U1; SECISBP2; SLC25A44; SLCO2B1; SPTLC2; SRRD; TAF4B; TNFRSF21; TMEM163; TRIM46; TRPT1; WLS; UROS; ZNF644

^a^BCO2, CREBRF; GPX3; HMG20A; JPH4; PAG1; SPTLC2 were also listed among the top 50 very important predictors (VIP) for residual feed intake (RFI), and IGF2, IRF2BP2, MACF1, MORC2, SDR39U1, TRIM46 and ZNF644 were listed among the top 50 VIP of feed conversion ratio (FCR)

## Discussion

Due to the integrative nature of FE and the difficulties to record it accurately for each pig, there is a strong need to identify relevant biomarkers of FE traits. Also, because transcriptomic differences in muscle and liver segregated pigs on RFI better than their genotype and farm of origin [24], we hypothesized that the landscape of gene expression levels in the whole blood, a compartment that summarizes the variations in tissue metabolism, may be used to find biomarkers of FE in growing pigs. The data presented herein confirmed that the gene expression profiling in the whole blood represented a relevant source to identify small sets of candidate biomarkers for two FE traits. Previous studies have identified about 1000 genes [1] and even more [2] that were differentially expressed in the whole blood between low and high RFI pig lines. But none have tried to identify molecular predictors for low/high RFI (binary classification) and quantitative values of FCR (prediction of individual values). For that, ML procedures have proven their capability to develop highly precise prediction models including FE [25]. In the current study, it was possible to discriminate pigs according to RFI (low/high) by using a subset of few blood transcripts (< 50) with 96 to 100% of success when using RF and GTB procedures, respectively. Moreover, it was possible to predict individual FCR, and not only the assignment of animals to divergent groups, by using another subset of 50 transcripts corresponding to 25 unique annotated genes with a good (~ 0.65; RF model) and very good (~ 0.80; GTB model) accuracy. Similarly, recent studies using ML algorithms [26, 27] in pigs showed that it was possible to predict the binary class of RFI by using the expression of 200 genes in the liver (accuracy: 0.78), 100 genes in duodenum (accuracy: 0.69) and 50 genes in skeletal muscle (accuracy: 0.61–0.70). In the current study, only 8% of all VIPs were identified as common predictors for RFI and FCR. These two traits are not equivalent, with only a moderate (0.39) genetic correlation between RFI and FCR [15], and in some studies, higher correlations between FCR and production traits than between FCR and RFI [28]. The common predictors in our study may correspond to the RFI part of FCR variability. Irrespective of the FE trait, the GTB procedures had better performance than the RF algorithms. This confirms that, despite a significant amount of overlap between the two methods and although RF performs well for class object detection, the gradient boosting methods result in better performance on other assessments like regression [22, 29]. Indeed, the GTB algorithm combines the gradient descent error minimization approach with boosting, and encapsulates an ensemble of weak prediction models added sequentially to improve the robustness of predictors [14]. In cattle, different ML methods have been tested to identify candidate genes for growth prediction, and the authors concluded that the better performance was obtained with the gradient boosting machine algorithm followed by the RF [30]. As compared with the individual ML method alone, combining RF and GTB together may further produce the highest value of prediction accuracy with the smallest subsets of genes that are biologically relevant to FE, as suggested in beef cattle [25]. This deserves further studies.

The subsets of genes combined in predictive models of FE in growing pigs were involved in several functional pathways that might be of equivalent importance in the definition of RFI and FCR. For some of them, they shared common transcriptional regulators. Finding relevant biological categories across the VIP attested to the reliability of the proposed candidates. Indeed, many genes in the immune/inflammatory system were identified as top predictors for RFI (*PSNE1, SERPINF1, MX1*), for FCR (*CD84*, *PIKFYVE, IRF2BP2)* and for both traits (*JPH4, PAG1*). This is consistent with findings that low RFI pigs had specificities in their immune tissue profile and capacity to respond to infectious or inflammatory challenges as compared with high RFI pigs [31, 32]. Especially, *IRF2BP2* (Interferon Regulatory Factor 2 Binding Protein 2) has emerged as an important transcriptional co-regulator in the immune system [33]. Moreover, *JPH4*, a gene that was also identified as differentially expressed in the liver of pigs that differed in FE [24], stimulates the expression of activation markers and cytokines [34]. However, *PAG1* negatively regulates T-cell activation [35]. Finding these two genes having opposite actions on T cell activation in the prediction model suggests subtle modifications in the regulation of immune signaling in pigs ranked for FE. Interestingly, *SERPINF1, HTRA1* and *NPR3* proposed for binary classification of pigs on RFI, have been previously identified as having the biggest changes in expression level in the whole blood between low and high RFI pig lines [3]. In accordance, Chen and colleagues [25] indicated that the GTB method picked up the top-ranked differentially expressed genes identified by *t*-test for FE in beef cattle. In the current study, some candidate biomarkers for FE were also associated with roles in the ubiquitination and protein modification process. The importance of the ubiquitin pathway may be over-estimated here, since we considered the whole blood where this process is specifically enriched [36]. This could be also related to the higher protein turnover identified in the liver of the most efficient pigs compared with less efficient pigs [31]. Because immunity, inflammation and ubiquitin-related protein modification are inter-related pathways, it is not surprising to find common genes in these pathways among the proposed biomarkers. For instance, *TRIM38* encoding the E3 ubiquitin ligase has multifaceted roles in innate immunity and inflammation [37]. Different genes related to antioxidant response and oxido-reduction activity, such as *GPX3* (glutathione peroxidase-3) and *BCO2* (beta-carotene oxygenase-2), were among the top-ranked VIPs for RFI classification. This is consistent with previous studies showing a difference in susceptibility to oxidative stress between low and high feed efficient pigs [38, 39]. Finally, lipid transport and catabolism, including *SPTLC2* and *MORC2* identified as top predictors, were underlined as biological pathways able to classify pigs according to RFI. Similarly, molecular alterations in lipid metabolism have been observed in the liver of low/high RFI pigs, having consequences on triglycerides, phospholipids or cholesterol concentrations in the blood of pigs from the same [3] or different [40] RFI lines. Among others, *PLA2G4A* (phospholipase A2 group IVA) identified as a common VIP for RFI and FCR traits, was recently suggested as a key regulator of fat deposition in chicken [41]. Increased circulating levels of IL-15 correlated with variations in adipose tissue mass and FCR in male mice fed a high-fat diet [42]. In the current study, it is unlikely that differences in diets between experimental pigs biased the results. Indeed, we did not observe any marked changes in the accuracy of prediction model for FCR reasoned on (net) energy intake or feed intake [26].

Altogether, this study reduced the complexity of FE into small subsets (< 50) of predictive transcripts. Among these candidate biomarkers for accurate predictions of RFI groups and FCR values, several genes have been already proposed as top molecular contributors to differences between low and high RFI lines, and even, in predictive models of FE. For instance, the expression level of *GPX3* was identified as affected by RFI selection in the whole blood [3], muscle, adipose tissues and liver of the same lines of pigs [16]. Similarly, *HTRA1* was listed as differentially expressed in both muscle and liver between low and high RFI pigs of two different populations [24]; this serine protease mediates multiple biological processes by antagonizing IGF-binding proteins and proteins of the TGF-beta family [43]. In line, the expression level of *IGF2* in muscle was identified as a reliable predictor for RFI breeding values [26] and increasing muscle growth through the IGF-1/2 signaling pathway was proposed as a potential strategy for the improvement of FE in Yorkshire pigs from 30 to 90 kg BW [44]. Expression level of *PSNE1* in muscle was also included in a predictive model for RFI breeding value in pigs of Large White breed [26]. The expression level of *SLC46A3* in liver, a gene involved in macromolecule degradation process [45], was identified as important to categorize pigs into RFI groups in the Hermitage Maxgro genotype [27]. Finding the same molecular candidates in different tissues is not surprising, since only < 10% of protein coding genes are tissue specific [46]. This reinforces the interest in using readily accessible samples in living animals such as blood to predict complex phenotypes. The proposed biomarkers can be also confronted to genomic regions identified by genome-wide association study (GWAS) as affecting FE traits. For instance, *TEX2* (Testis Expressed 2 protein) identified here as a predictive biomarker for RFI classification, was identified as a positional candidate in SNP detected for average daily feed intake (a FE related trait) in Landrace pigs [47].

## Conclusion

This study identified small sets of transcripts in the whole blood as candidate biomarkers for FE traits, namely RFI group (low/high) and FCR values measured in growing pigs. Since nutritional requirements for the most efficient pigs might be slightly greater than usual feed recommendations [48], these circulating biomarkers could be further used as a decision support tool for feeding animals with an appropriate diet. Therefore, this study offers encouraging perspectives for assigning animals to phenotypic groups and to be used as proxy of FE in large numbers of animals. Additional studies are required to confirm the generality of the predictions in other pig breeds and crossbreds before these circulating biomarkers could be readily used for precision farming strategies.

## Methods

### General design

This study reused phenotypic data obtained in pigs from the three independent experiments that were previously published [19–21], to avoid the needs of new sampling in living animals while obtaining a high number of animals allowing robust predictions. The application of ML procedures on the merged dataset (*n* = 148 pigs) avoided the overfitting often observed when simple classification or regression procedures are used for a limited number of animals and a high number of dependent variables, and the leave-one-out method was an additional way to resampling the datasets. Thus, this study fits with the 3R (Replacement, Reduction and Refinement) principles.

### Pigs and blood samples

The three independent datasets referred to purebred French Large White pigs produced in a divergent selection experiment for RFI. The selection program was described in full details elsewhere [49], including the equation to calculate RFI from a regression between observed feed intake and that expected based on requirements for maintenance (based on the metabolic BW) and performance (average daily gain, backfat thickness). From birth to weaning, all pigs were reared in the selection farm of INRAE (UE Genesi, Le Magneraud & Rouillé, France; 10.15454/1.5572415481185847E12). All pigs were weaned at 28 days (d), and were first fed ad libitum with standard starter and weaner diets. During subsequent test periods in dedicated buildings, pigs have undergone different feeding conditions depending on the experiments as described below. As indicated in the referenced publications [19–21], the three experiments were conducted in accordance with the French legislation on animal experimentation, and the protocols were approved by regional ethical committees evaluating the research question, design, plan analysis, animal care and monitoring, and ways to minimize pain and consider limit points (especially regarding jugular blood sampling). At the end of each experiment, pigs were slaughtered using approved procedures, including electronarcosis followed by jugular exsanguination.

The first dataset [19] included 21 castrated males from the 7th generation of selection (*n* = 10 low RFI pigs and *n* = 11 high RFI pigs) housed at thermo-neutrality (24 °C) and reared at the INRAE experimental pig facility at Saint-Gilles, France (UE3P, 10.15454/1.5573932732039927E12). At 80 d of age, pigs were transferred in individual cages, and were fed a standard diet that met nutritional requirements for growth. At 87 d of age (59.2 kg BW on average), blood was collected from the jugular vein and prepared for RNA extraction. The feed conversion ratio (FCR) was calculated from individually measured daily feed intake and average daily gain for the 14 d of the trial (i.e., from 87 d to 100 d of age).

The second dataset [20] included 48 castrated males from the 8th generation of selection (*n* = 24 low RFI pigs and *n =* 24 high RFI pigs). Pigs were reared at the INRAE experimental pig facility at Saint-Gilles, France (UE3P, 10.15454/1.5573932732039927E12). At 74 d of age, pigs were transferred in individual cages and after 2 d of transition, the first half was fed a standard diet and the second half was fed a high-fiber high-fat diet during the growing and finishing phases. At 132 d of age (average BW of 75.6 kg), blood was sampled from the jugular vein and prepared for RNA extraction. The FCR was then calculated from 76 d to 132 d of age.

The third dataset [21] included 79 castrated males and females from the 9th generation of selection (*n* = 37 low RFI pigs and *n* = 42 high RFI pigs). Pigs were reared at the experimental INRAE pig facility at Le Magneraud, France (UE Genesi; 10.15454/1.5572415481185847E12). Blood was sampled at 40 d of age from the jugular vein. At 70 d of age, pigs were transferred to group-housing facilities equipped with single-place electronic feeders. The first half of the pigs were fed standard diets, whereas the second half was fed a high-fiber diet during the growing-finishing phases [21]. The FCR was then calculated from 90 d to 161 d of age.

In the three datasets, the reference to low or high RFI line was indicated for each pig, and the FCR value was individually attributed. Other factors (sex, season, generation, diet) were not taken into account.

### Microarrays data

Microarray data considered in the current study were obtained from the referenced publications in the first [19] and second [3] experiments, and were newly acquired from RNA extracted from the stored blood samples in the third experiment. All experiments followed the same procedures for RNA extraction and expression data generation. The porcine commercial Agilent-026440 microarray (V2, 44 K, GPL15007, Agilent Technologies, Massy, France) had been used in the first experiment (representing about 12,332 unique annotated genes). The custom porcine microarray (8x60K, GPL16524 Agilent Technologies) that contained the same probes as the Agilent-026444 and an additional set of probes enriched with immune system, muscle and adipose tissue genes, has been used in the second and third experiments (representing about 14,466 unique annotated genes). In the three transcriptomic datasets, raw spot intensities have been submitted to quality filtration based on four criteria: background intensity value, diameter, saturation and uniformity of the spot, and intensities of filtered spots were log2 transformed and median-centered to correct for microarray effect.

For the current study, the three microarray datasets were then merged into a single new dataset. There was no exclusion of any animals in this merged dataset. To obtain consolidated expression values across the three independent datasets, the molecular data have been normalized by mean centering, i.e. subtracting the mean value across all probes from all raw values for each pig sample in the merged dataset. The merged dataset also included meta-data such as the experiment of origin (1, 2, and 3), RFI group (*n* = 71 pigs of low RFI line, *n* = 77 pigs of high RFI line) and FCR value (*n* = 148 pigs). All data were deposited in a publicly available repository at 10.15454/J4XOPD.

### Supervised methods to identify important variables for the prediction of FE traits

The merged dataset was used to search the most important molecular predictors for the RFI group and FCR value, by using ML methods. The experimental unit was the pig. Among the panel of ML methods for dimensionality reduction, classification and regression used in livestock breeding [14], the RF and GTB procedures were chosen in the current study and were compared for performance in classification (RFI group) and regression (FCR value) procedures. These two ML methods use decision trees, but RF uses a large number of trees combined by averaging or “majority rules” at the end of the process [50], whereas GTB starts the combining process of decision trees at the beginning [27, 51, 52]. Other differences include how trees are built: RF builds each tree independently, while GTB builds one tree at a time but in an additive model proceeding in a forward stage-wise sequential error–correcting process to combine results along the way and converge to an accurate model [29]. Sequential steps for learning, validation, and finally, selection of the best models were performed according to standards described by Fernandez-Lozano and colleagues [53]. Models were generated from RF and GTB algorithms with Salford Predictive Modeler 8.0 (SPM 8.0®).

The RF models were generated with about 1500 trees for classification of RFI and regression for FCR. For that, a randomly selected bootstrap sample set was created by using 50% of the original dataset for learning (*n* = 74 pigs). Consequently, each bootstrap sample called “out-of-bag” data (OOB) excluded 50% of the data that were further used for validation (*n =* 74 pigs), and the leave-one-out method assessed the performance by resampling the training set. The test dataset allowed cross-validation ensuring that the training of the model was not biased. To split branches of a tree, a random sample of m variables was chosen from the full set of p variables. The partition of probes between learning and validation datasets was shown in Supp. Fig. S2. We checked that the three experiments of origin were included in both the training and validation datasets.

The GTB prediction models were also generated using 1500 small decision trees for classification or regression, and using a randomly selected bootstrap sample set for learning (*n =* 74 pigs) and the remaining data (*n =* 74 pigs) for validation. As recommended, each tree typically contained about six terminal nodes. The model was like to Fourier or Taylor series, which is a sum of factors that becomes progressively more accurate as the expansion continues. After each step of boosting, the algorithm scaled the newly added weights, which balanced the influence of each tree. The accuracy of the algorithm was improved by introducing randomization through training the base learner on different randomly selected samples at each iteration.

In both procedures, significant variables were selected using the Gini index to evaluate the discriminant ability of the potential selected feature, defined as:

$$ \mathrm{Gi}=1-\sum jp2\ \left(j|t\right) $$

Where p2 (*j* | *t*) is the estimated class probability for feature *t* or node *t* in a decision tree and *j* is an output data or class. Only the variables that improved Gini index and minimized the OOB error rate were retained as very important variables in prediction (VIP).

Multiple runs for each ML methods were performed (ten times) to consider variations in the observations used for the training step (using permutations and leave-one-out procedures) and the stability of the techniques. The iteration steps were also applied to reduce the number of VIPs in the selected models. At each run, the accuracy of classification models was estimated with the proportion (%) of good classification and the optimal models were selected according to the ROC curve. In regression, RMSE was calculated as the square root of the difference between the realized and the predicted observation within the OOB data after permuting each predictor variable in the training dataset divided by the number of trees for the regression procedure. The adjusted coefficient of determination (*R*^2^) was also computed. The predicted (X) values for FCR obtained by the best GTB model and the observed (Y) values measured on the pigs were compared (X-Y) using the GLM procedure. The model was considered unbiased when the intercept obtained by the GLM model was not different from 0 and the slope was not significantly different from 1. The quality of the relationships was evaluated based on RMSE of prediction (RMSEP) obtained by a leave-one-out cross-validation from the value of the predicted residual sum of squares.

### Pathway enrichment analysis

Gene-annotation enrichment analyses among the VIP identified for binary classification of pigs on RFI and prediction of FCR were performed on encoded genes by using DAVID bioinformatics tool on default settings [54].

## Supplementary Information

**Additional file 1: Supp. Table S1** Iterative steps for model reduction to predict RFI class using different machine learning algorithms

**Additional file 2: Supp. Table S2** List of probes identified as important to classify pigs in low or high RFI using random forest algorithm on transcripts levels of genes in the whole blood

**Additional file 3: Supp. Fig. S1** Regression analysis of the relationship between observed and predicted FCR according to the dataset of origin Partition of pigs used in the test dataset for validating

**Additional file 4: Supp. Fig. S2** Partition of molecular probes expressed in the whole blood between trained and validation datasets to analyze traits related to feed efficiency in pigs

## Data Availability

The datasets generated and analyzed in the current study are deposited in a publicly available repository at 10.15454/J4XOPD
