# Supplementary material for: Analysis of merged whole blood transcriptomic datasets to identify circulating molecular biomarkers of feed efficiency in growing pigs
Source: BMC Genomics. 2021 Jul 3;22:501. doi: 10.1186/s12864-021-07843-4 (PMC8254903; doi:10.1186/s12864-021-07843-4)
Supplement: Supplementary file 1 — Additional file 1: Supp. Table S1 Iterative steps for model reduction to predict RFI class using different machine learning algorithms [file 12864_2021_7843_MOESM1_ESM.docx]

**Supp. Table S1** Iterative steps for model reduction to predict RFI class using different machine learning algorithms

| Models | Nb VIP | Average (%) | Overall Correct (%) | ROC OOB | K-S Lean |
| --- | --- | --- | --- | --- | --- |
| Random Forest | 328 | 95.98 | 95.95 | 0.989 | 0.919 |
|  | 100 | 95.98 | 95.95 | 0.993 | 0.947 |
|  | 50 | 97.46 | 97.47 | 0.993 | 0.945 |
|  | 25 | 97.30 | 97.30 | 0.996 | 0.945 |
|  | 10 | 97.30 | 97.30 | 0.996 | 0.945 |
| Gradient Tree Boosting | 391 | 100 | 100 | 1 | 1 |
|  | 100 | 100 | 100 | 1 | 1 |
|  | 50 | 100 | 100 | 1 | 1 |
|  | 25 | 100 | 100 | 1 | 1 |
|  | 10 | 100 | 100 | 1 | 1 |

Random forest (RF) and gradient tree boosting (GTB) algorithms were applied on a transcriptomic dataset containing 26,687 molecular probes measured in whole blood sampled from 148 pigs. Dataset was split into training (n=74) and validation test (n=74) subsets to evaluate models performance in classifying pigs into low or high residual feed intake (RFI) groups. Success rate (%) was evaluated for different iterative steps used to reduce the initial dataset into the most relevant probes (so called very important variables in prediction, VIP) able to attribute the right class for each pig. Whatever the number of retained VIP, the rate of success was better with the GTB procedure than with the RF algorithm.
