# Supplementary material for: Analysis of merged whole blood transcriptomic datasets to identify circulating molecular biomarkers of feed efficiency in growing pigs
Source: BMC Genomics. 2021 Jul 3;22:501. doi: 10.1186/s12864-021-07843-4 (PMC8254903; doi:10.1186/s12864-021-07843-4)
Supplement: Supplementary file 2 — Additional file 2: Supp. Table S2 List of probes identified as important to classify pigs in low or high RFI using random forest algorithm on transcripts levels of genes in the whole blood [file 12864_2021_7843_MOESM2_ESM.docx]

**Supp. Table S2** List of probes identified as important to classify pigs in low or high RFI using random forest algorithm on transcripts levels of genes in the whole blood

| Probe name | Gene symbol | Full name | Score |
| --- | --- | --- | --- |
| A_72_P304024 | PSEN1 | presenilin 1 | 24.11 |
| A_72_P359418 | WDHD1 | WD repeat and HMG-box DNA binding protein 1 | 4.56 |
| A_72_P418319 | HTRA1 | HtrA serine peptidase 1 | 3.61 |
| A_72_P763826 | CYP24A1 | cytochrome P450, family 24, subfamily A, polypeptide 1 | 3.55 |
| A_72_P742047 | SERPINF1 | serpin peptidase inhibitor, clade F (alpha-2 antiplasmin, pigment epithelium derived factor), member 1 | 3.52 |
| O12841 | PARVG | parvin, gamma | 3.29 |
| A_72_P387418 | C6orf221 | chromosome 6 open reading frame 221 | 2.67 |
| A_72_P008221 | SERPINF1 | serpin peptidase inhibitor, clade F (alpha-2 antiplasmin, pigment epithelium derived factor), member 1 | 2.66 |
| A_72_P723043 | SERPINF1 | serpin peptidase inhibitor, clade F (alpha-2 antiplasmin, pigment epithelium derived factor), member 1 | 2.61 |
| A_72_P146401 | SERPINF1 | serpin peptidase inhibitor, clade F (alpha-2 antiplasmin, pigment epithelium derived factor), member 1 | 2.60 |
| O12773 | PCIF1 | PDX1 C-terminal inhibiting factor 1 | 1.97 |
| A_72_P548816 | HMG20A | high mobility group 20A | 1.93 |
| A_72_P337333 | CD1A | CD1a molecule | 1.79 |
| A_72_P250342 | RPS18 | ribosomal protein S18 | 1.79 |
| A_72_P035801 | EPAS1 | endothelial PAS domain protein 1 | 1.63 |
| A_72_P585246 | PCIF1 | PDX1 C-terminal inhibiting factor 1 | 1.45 |
| A_72_P633086 | CD1A | CD1a molecule | 1.40 |
| A_72_P185296 | CLU | Clusterin | 1.39 |
| O12605 | HMG20A | high mobility group 20A | 1.17 |
| A_72_P131741 | SLC46A3 | solute carrier family 46, member 3 | 1.16 |
| A_72_P006091 | PLA2G4A | phospholipase A2, group IVA (cytosolic, calcium-dependent) | 1.14 |
| A_72_P609509 | CD1A | CD1a molecule | 0.88 |
| O8180 | ARID3B | AT rich interactive domain 3B (BRIGHT-like) | 0.76 |
| A_72_P121746 | SLCO2B1 | solute carrier organic anion transporter family, member 2B1 | 0.75 |
| A_72_P177616 | DCT | dopachrome tautomerase (dopachrome delta-isomerase, tyrosine-related protein 2) | 0.74 |
| A_72_P671275 | GPX3 | glutathione peroxidase 3 (plasma) | 0.72 |
| A_72_P440086 | GPX3 | glutathione peroxidase 3 (plasma) | 0.59 |
| O10865 | BMPR2 | bone morphogenetic protein receptor, type II (serine/threonine kinase) | 0.55 |
| A_72_P337268 | HEATR4 | HEAT repeat containing 4 | 0.54 |
| A_72_P473804 | WWP1 | WW domain containing E3 ubiquitin protein ligase 1 | 0.49 |
| O12495 | TM7SF2 | transmembrane 7 superfamily member 2 | 0.45 |
| A_72_P000006 | ZNF644 | zinc finger protein 644 | 0.42 |
| A_72_P570814 | ATP5O | ATP synthase, H+ transporting, mitochondrial F1 complex, O subunit | 0.40 |

A random forest (RF) algorithm was applied on transcriptomic dataset (26,687 molecular probes) from the whole blood of 148 pigs. Data were split into training (n=74) and validation test (n=74) subsets to evaluate model performance in predicting feed conversion ratio (FCR). A subset of 50 molecular probes were retained by the algorithm as important for FCR prediction with a good accuracy (R²=0.80; RMSE=0.23; RMSEP=0.15). Corresponding identified genes were listed by the order of importance (score).
