# Supplementary material for: Analysis of merged whole blood transcriptomic datasets to identify circulating molecular biomarkers of feed efficiency in growing pigs
Source: BMC Genomics. 2021 Jul 3;22:501. doi: 10.1186/s12864-021-07843-4 (PMC8254903; doi:10.1186/s12864-021-07843-4)
Supplement: Supplementary file 3 — Additional file 3: Supp. Fig. S1 Regression analysis of the relationship between observed and predicted FCR according to the dataset of origin Partition of pigs used in the test dataset for validating [file 12864_2021_7843_MOESM3_ESM.docx]

**Supp. Fig. S1** Regression analysis of the relationship between observed and predicted FCR according to the dataset of origin Partition of pigs used in the test dataset for validating

The graph was computed between observed and predicted feed conversion values (FCR). The black dots represent pigs of the first dataset, the red dots represent pigs of second dataset and the green dots represent pigs of the third dataset.
