# Supplementary material for: Analysis of merged whole blood transcriptomic datasets to identify circulating molecular biomarkers of feed efficiency in growing pigs
Source: BMC Genomics. 2021 Jul 3;22:501. doi: 10.1186/s12864-021-07843-4 (PMC8254903; doi:10.1186/s12864-021-07843-4)
Supplement: Supplementary file 4 — Additional file 4: Supp. Fig. S2 Partition of molecular probes expressed in the whole blood between trained and validation datasets to analyze traits related to feed efficiency in pigs [file 12864_2021_7843_MOESM4_ESM.docx]

**Supp. Fig. S2** Partition of molecular probes expressed in the whole blood between trained and validation datasets to analyze traits related to feed efficiency in pigs


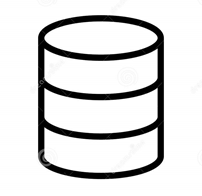


**Training data sets (3 merged data sets)**

**26,687 probes**

**N =74 pigs**


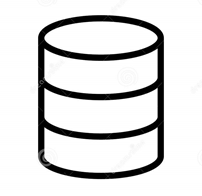


**Validation data sets (RFI)**

**778 probes**

**N =74 pigs**


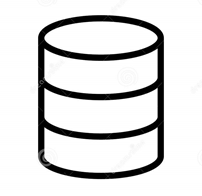


**Validation data sets (FCR)**

**1,393 probes**

**N =74 pigs**

Three microarrays dataset generated from the whole blood of 148 growing pigs were merged into a single dataset (26,687 expressed annotated probes). Randomly selected bootstrap pig samples (n = 74) were used for training, whereas the remaining pig samples (n = 74) were used for validation. Subsets of molecular probes (<5% of the all probes) were selected as important to predict class of residual feed intake (RFI) and value of feed conversion ratio (FCR) by using machine learning algorithms.
